# Iron Deficiency Anemia Presenting With Pancytopenia: A Study From India

**DOI:** 10.7759/cureus.45034

**Published:** 2023-09-11

**Authors:** Mahadev Meena, Satyendra Khichar, Akash Pawar, Naresh Midha, Saurabh Kumar, Abhishek Purohit, Gopal K Bohra, Mahendra Kumar Garg, Abhishek Singhai

**Affiliations:** 1 General Medicine, All India Institute of Medical Sciences, Bhopal, Bhopal, IND; 2 General Medicine, All India Institute of Medical Sciences, Jodhpur, Jodhpur, IND; 3 Neurology, King George's Medical University (KGMU), Lucknow, IND; 4 Pathology and Hematopathology, All India Institute of Medical Sciences, Jodhpur, Jodhpur, IND

**Keywords:** iron deficiency anemia, hemoglobin electrophoresis, direct agglutination test, lactate dehydrogenase (ldh), pancytopenia

## Abstract

Introduction: Iron deficiency anemia (IDA) is the most common cause of anemia worldwide. IDA is commonly associated with thrombocytosis and normal or slightly decreased leukocyte count. Sometimes it can present with thrombocytopenia, but rarely present with pancytopenia. Here we are presenting six cases of severe iron deficiency presenting with pancytopenia, which responded to iron replenishment.

Methods: This 12-month observational study was conducted in the Department of General Medicine at a tertiary care Centre in India. All cases of pancytopenia (after exclusion of other causes) with IDA were included. IDA was established with the help of a complete blood count (CBC), peripheral smear examination, serum iron studies, and serum ferritin.

Results: In our study, CBC at four weeks later of iron transfusion without other supplementation showed significant improvement in hematological parameters.

Conclusion: Severe iron deficiency is a reversible etiology of pancytopenia. It should be kept as a differential diagnosis of pancytopenia if common causes of pancytopenia are ruled out.

## Introduction

Iron deficiency anemia (IDA) is the most common cause of anemia worldwide [1]. It is defined as a decrease in iron stored in the body, which is measured by serum ferritin level. In developing countries, IDA is rather common owing to low dietary intake, poor availability of iron in the diet, chronic inflammation, and chronic blood loss due to hookworm infestation or hemorrhoids [2]. IDA is commonly associated with thrombocytosis and normal or slightly decreased leukocyte count [3-4]. Sometimes it can present with thrombocytopenia, but pancytopenia is rarely seen [5-6]. Pancytopenia is a clinical condition characterized by anemia, leukopenia, and thrombocytopenia. The cause of pancytopenia can be bone marrow failure, peripheral destruction, or sequestration of blood cells. Underlying etiology determines the management and prognosis of patients with pancytopenia. Severe iron deficiency is a rare but reversible cause of pancytopenia with an excellent prognosis. In this study, we are presenting six cases of severe iron deficiency presenting with pancytopenia, which improved with iron replenishment. Our objective was to describe the relatively uncommon finding (pancytopenia) in an extremely common iron deficiency anemia.

## Materials and methods

This observational prospective study was conducted in the Department of General Medicine of All India Institute of Medical Sciences, Jodhpur, a tertiary care center in India. This was an observational study conducted over a 12-month duration. Cases were inpatients that presented with anemia and were found to be pancytopenia on evaluation by complete blood count (CBC) and peripheral blood film (PBF). According to the World Health Organization, pancytopenia was defined as hemoglobin (Hb)<12 gm/dL for non-pregnant women and <13 gm/dL for men, total leukocyte count (TLC) less than 4000/mm^3^, platelet counts of <150,000/mm^3^. We took patients with Hb less than 10 gm/dL, TLC less than 4000/mm^3^, platelet counts less than 100,000/mm^3^. All the cases of pancytopenia (after exclusion of other causes) with IDA were included. IDA was established with the help of CBC, peripheral smear examination, serum iron studies, and serum ferritin. On further investigations, the peripheral blood picture showed microcytic hypochromic anemia with pancytopenia. Iron deficiency was defined by serum ferritin level <30 µg/L, serum iron <30 µg/dL (normal range for males: 80-180 µg/dL; normal range for females: 60-160 µg/dL), and total iron binding capacity (TIBC) >360 µg/dL. Other common causes of pancytopenia like vitamin B_12_ and folic acid deficiency (serum B_12_, folate level), aplastic anemia (bone marrow aspiration and biopsy), hematological malignancies, malaria, autoimmune diseases (anti-nuclear antibody), and hypersplenism were ruled out. Complete clinical details (presentation, duration of illness, occupation, treatment, and addiction) and details of laboratory investigations (hematological as well as biochemical investigations, CBC, PBF) were collected and analyzed. The study's objectives, methods, risks, and benefits were explained to patients prior to their participation. We provided this information both verbally and in a written document in their language. We assured the participants that their participation was completely voluntary and would not affect their medical care. Informed written consent was obtained from all the participants. During the whole study, patient confidentiality was maintained.

Microsoft Excel (Microsoft Corporation, Redmond, USA) was used to enter the information gathered from the study participants'^ ^medical records into a computerized database. In this study, all patients were female, and the age range was 30-46 years. Mean age was 39.16 years. The mean Hb before treatment was 4.8 gm/dL and 10.25 gm/dL after treatment. In this study, mean serum iron and ferritin were 8.33 µg/dL and 8.56 µg/L, respectively.

## Results

Case 1: A 35-year-old female presented with a complaint of generalized weakness and low-grade fever associated with pedal edema. On investigations, she^ ^was found to have pancytopenia (Table 1). Peripheral blood smear denoted hypochromic microcytic anemia. Her blood lactate dehydrogenase (LDH) was 231 units/L, and B_12_ was 514mcg/L. Stool occult blood and direct agglutination test (DAT) were negative. Her Hb electrophoresis did not show abnormal Hb (A1, 97.8%; A2, 1.68%; F, 0.52%).

**Table 1:** Summary of patients finding (before and after treatment) Hb, hemoglobin; TLC, total leukocyte count; PLT, platelets; MCV, mean corpuscular volume; RETI, reticulocyte count; TIBC, total iron binding capacity; ANA, anti-nuclear antibody; Y/F, years/female

Parameters	Normal value	Case no. with age and sex											
		Before iron transfusion						After four weeks of iron transfusion					
		1 (35 Y/F)	2 (45 Y/F)	3 (46 Y/F)	4 (37 Y/F)	5 (42 Y/F)	6 (30 Y/F)	1 (35 Y/F)	2 (45 Y/F)	3 (46 Y/M)	4 (37 Y/F)	5 (42 Y/F)	6 (30 Y/F)
Hb (g/dL)	11-15 (g/dL)	5.9	4.1	5	4.4	6.8	3.1	11.4	9.3	12.15	9.4	9.2	10.1
TLC (/cumm)	4-^24^11x1000/cumm	2050	3770	2160	3080	2530	2710	5743	7122	6140	5432	5900	7432
PLT (/cumm)	1.5-4.5 lakhs^25 ^/cumm	41000	30000	46000	90000	99000	68000	192000	^26^42000	234000	210000	143000	239000
MCV (fL)	76-93 fL	68.2	56.0	63.8	64.9	58.9	62.7	78.9	69.4	80.4	73.5	75.8	76.3
RETI (%)	0.82-2.25%	2.11	2.10	1.29	1.45	1.23	2.34	4.83	4.03	2.19	3.30	3.42	4.45
Serum iron (µg/dL)	60-180 (µg/dL)	08	07	05	08	09	13						
Serum ferritin (µg/L)	24-307 (µg/L)	20.3	5.2	5.9	11.4	2.30	6.3						
TIBC (µg/dL)	240-450 (µg/dL)	456	462	375	366	449	551						
ANA		Negative	Negative	Negative	Negative	Negative	Negative						

As a part of the workup of pancytopenia, a bone marrow examination was done. Bone marrow was cellular and revealed all the hematopoietic components. Erythroid maturation was micronormoblastic and other components were of normal morphology. In view of low serum iron and ferritin, 1437 mg of total iron dose was infused in the form of ferric carboxymaltose. The patient improved after iron supplementation as reticulocyte count on day seven was 5.06%. CBC also showed significant improvement at four weeks of iron transfusion (Table 1). Thrombocytopenia improved in our patients at three months follow-up.

Case 2: A 45-year-old female presented with a one-month history of shortness of breath on exertion, which worsened over time. Laboratory investigations (Table 1) suggested pancytopenia with microcytic hypochromic red cell picture in peripheral blood smear. The patient’s vitamin B_12_ (314 mcg/dL) and serum LDH (185 units/L) were normal. DAT and stool occult blood test results were negative. Hb electrophoresis demonstrated A1: 96.9%, A2: 2.3%, and F: 0.8%.

The patient was transfused one unit of packed red cells for symptomatic relief. Iron studies revealed severe iron deficiency; hence the patient was administered 1675 mg in the form of ferric carboxymaltose. The patient showed hematological improvement by the end of the first week (reticulocyte count: 4.98%) after iron infusion, which was continued in the next follow-up at four weeks (Table 1).

**Figure 1:**
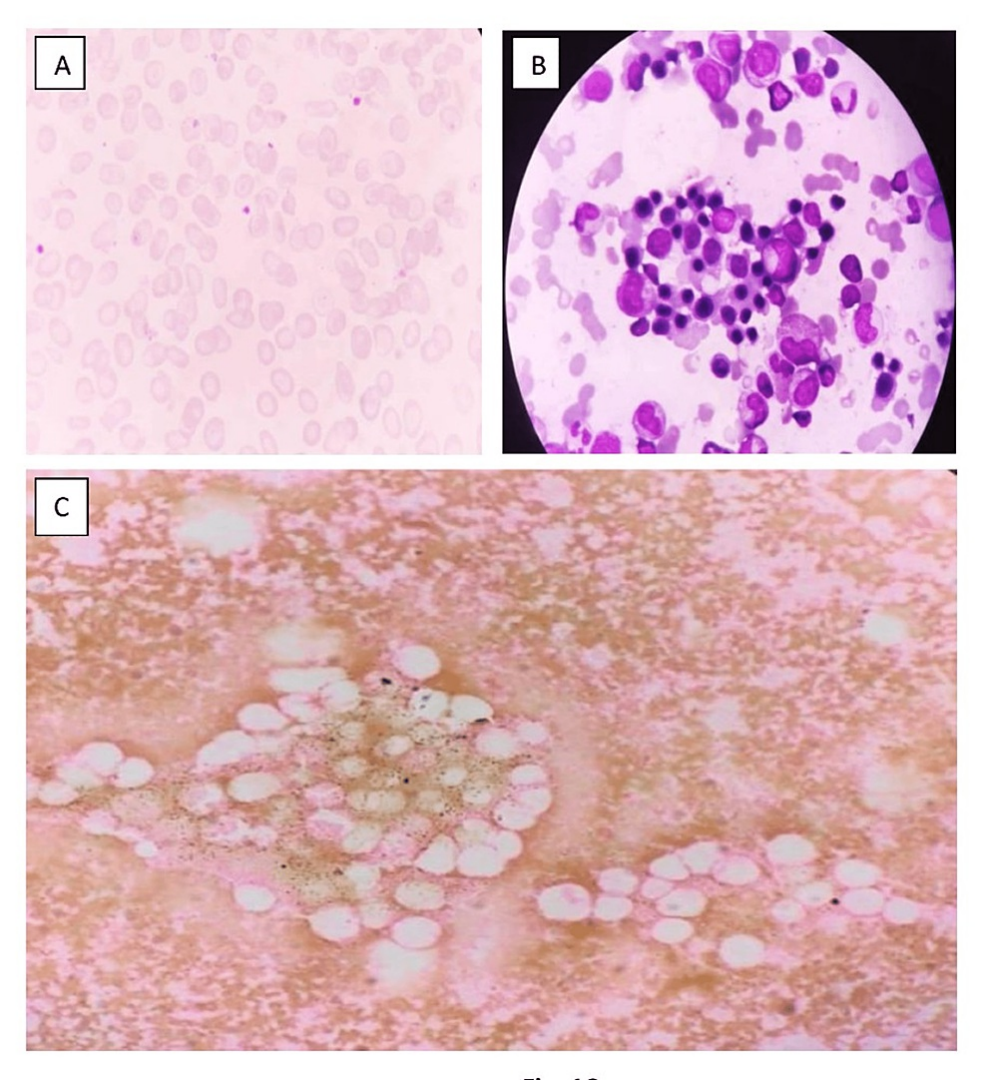
Case 3: Blood film morphology and bone marrow aspiration A: PBS, Leishman stain. 100x shows microcytic hypochromic cells with few target cells and elliptical cells; B: BMA, Giemsa stain. 100X shows erythroid prominence with micronormoblastic reaction; C: BMA, Prussian blue stain. 40X shows a complete lack of iron stores. PBS, peripheral blood smear; BMA, bone marrow aspiration

Case 3: A 46-year-old female with a known case of chronic liver disease with portal hypertension presented with a three-day history of fever, dry cough, and dizziness.^ ^The physical examination revealed massive splenomegaly. Laboratory investigation (Table 1) suggested pancytopenia. Blood film morphology and bone marrow aspiration (Figure 1) were suggestive of an iron-deficient state confirmed by an iron study (Table 1). Her blood LDH and vitamin B_12_ were 300 units/L and 756 mcg/dL. DAT was negative, and the stool occult blood test was positive. Upper gastrointestinal (UGI) endoscopy demonstrated grade 3 esophageal varices, and endoscopic band ligation was done.

The patient was administered iron supplementation, and the patient gradually started responding in the form of increasing reticulocyte count (3.89%) on day seven. After four weeks, her Hb was 12.1 gm/dL, TLC was 6740/cumm, and platelet count was 234,000/cumm.

Case 4: A 37-year-old female, a suspected case of extrahepatic portal vein thrombosis with chronic liver disease and portal hypertension, presented with complaints of whole pain abdomen, vomiting, loose stool, and fever for the last ten days. Laboratory reports suggested pancytopenia, and blood film morphology revealed moderate anisopoikilocytosis, microcytic hypochromic occasional fragmented cells, and pencil cells with mild rouleaux formation; TLC and platelet were reduced on the smear. Bone marrow was cellular with the entire hematopoietic component. Her vitamin B_12_ and serum LDH were 388 mcg/dL and 303 units/L, respectively. The stool occult blood test was positive. Endoscopic band ligation of esophageal varices was done as demonstrated in UGI endoscopy. 

Her iron profile (Table 1) is suggestive of iron deficiency and she started to show a response to intravenous iron supplementation. The total infused dose of iron was 1635 mg in the form of ferric carboxymaltose. At day seven, the reticulocyte count was 3.40%. After four weeks of iron transfusion, her Hb was 9.4 g/dL, TLC was 5432/cumm, and platelet was 210000/cumm.

Case 5: A 42-year-old female presented with complaints of generalized weakness and exertional dyspnoea for two months, which was progressively deteriorating. On examination, the patient had pallor with no other significant finding. The lab report was suggestive of pancytopenia (Table 1). Blood film morphology showed microcytic hypochromic red blood cells (RBC) with occasional fragmented cells, pencil cells, tear drop cells, leukopenia, and thrombocytopenia. Indirect and direct agglutination tests and stool occult blood tests were negative with normal Hb electrophoresis. Blood LDH was 189 units/L, and B_12_ was 502 mcg/dL. The patient’s serum ferritin level was reduced (2.39 mcg/L). The total infused iron dose was 1318 mg in the form of carboxymaltose. The patient’s reticulocyte count after seven days of iron transfusion was 5.09% and improved gradually with injectable iron supplementation.

Case 6: A 30-year-old female presented with complaints of insidious and gradually progressive exertional dyspnea. On examination, she had pallor with no other significant finding. Blood film morphology showed predominantly microcytic hypochromic RBC, marked anisopoikilocytosis, tear drop cells, pencil cells, target cells, fragmented RBCs and polychromatophils, slightly reduced TLC, and thrombocytopenia. Her vitamin B_12_ and serum LDH were 1750 mcg/dL and 156 units/L, respectively. Hb electrophoresis was normal.

The iron profile (Table 1) is suggestive of iron deficiency, and she started to show a response to intravenous iron supplementation in the form of increased reticulocyte count (5.80%) at day seven. After four weeks of iron supplementation, Hb was 10.1, TLC was 7432/cumm, and platelet was 239000/cumm.

## Discussion

Pancytopenia is routinely encountered and is an important hematological entity in India. The etiology of pancytopenia varies according to geography and genetics [7-10]. Aplastic anemia is the common cause of pancytopenia throughout the world as reported in various studies [8]. This is diametrically different from the results of most Indian studies, where the most common cause of pancytopenia was megaloblastic anemia [10-12]. Megaloblastic anemia, which is the common cause of pancytopenia, stresses the higher prevalence of nutritional anemia in the Indian scenario that is correctable and avoidable [10-12]. IDA is a common disease in developing countries. It usually presents with anemia and thrombocytosis. Pancytopenia is a rare hematological presentation of Iron deficiency.

In this study, patients with iron deficiency presented with pancytopenia. On evaluation, they showed a hematological picture of severe IDA. Other common causes of pancytopenia like vitamin B_12_ and folic acid deficiency, aplastic anemia, hematological malignancies, malaria, autoimmune diseases, and hypersplenism (bone marrow and improvement after iron supplementation) were ruled out. Two patients received a blood transfusion for treatment of symptomatic anemia. All patients are treated with intravenous iron replacement therapy followed by oral iron supplements. These resulted in improvement in the platelet and TLCs and finally complete recovery of the whole blood picture.

Usually, IDA presents as microcytic hypochromic anemia often with reactive thrombocytosis without much involvement of other hematological elements but other hematological parameters may be affected occasionally [13]. Iron deficiency sometimes presents with thrombocytopenia and rarely as pancytopenia [5-6]. The mechanism behind thrombocytopenia is unclear, but it may be due to an alteration in the activity of iron-dependent enzymes in thrombopoiesis or early response to direct stimulation of the erythropoietin (EPO) receptor on megakaryocytes or shunting into the erythroid precursors' pathway [14-15].

The mechanism of leukopenia seen in iron deficiency is not clear. However, it can be explained by the down-regulation of neutrophil production by a high level of EPO as it occurs in animal studies or alteration in iron-dependent enzyme activity in thrombopoiesis and leucopoiesis [6,14].

In their study, Osama Ishtiaq et al. found IDA as the fourth most common cause of pancytopenia [16]. Anita et al. also reported 13 cases in which IDA was the second most common cause of pancytopenia [17]. In a study done by Tilak and Jain, megaloblastic anemia (68%) was the common cause of pancytopenia followed by aplastic anemia (7.7%) [8]. This study also revealed a few uncommon and rare but interesting causes of pancytopenia like iron deficiency. In a study of 251 patients by Varma et al., the common cause of pancytopenia was megaloblastic anemia (39%) followed by dimorphic anemia (14%) and aplastic anemia (7%). IDA was the fourth etiology of pancytopenia in 5.9% (15/251) cases [18]. In another Indian study, IDA was the third common cause of pancytopenia and 70% of cases were treatable with complete recovery from pancytopenia [19]. In case reports by Karami H et al., two young boys presented with weakness, lethargy, fatigue, and pallor without organomegaly and lymphadenopathy. On further evaluation, they found pancytopenia. Peripheral blood smears of both patients showed hypochromic microcytic RBCs with some teardrop cells and ovalocytes. Bone marrow aspiration was cellular marrow without blast and abnormal cells. DAT was negative and Hb electrophoresis was normal; however, both patients’ serum ferritin was decreased (Case 1: 0.9 ng/mL; Case 2: 2 ng/mL). Other iron profile parameters also favored IDA and patients responded well with oral iron [20]. In another case report, a seven-year-old male child presented with pancytopenia and was diagnosed as IDA, without other causes of pancytopenia or any other malignant disease. He received a packed cell transfusion in view of symptomatic anemia. His blood parameters were improved with the starting and continuation of iron supplements [21]. In another Indian study, the most common etiology of pancytopenia was megaloblastic anemia (34%), and iron deficiency anemia was the fourth cause (8%). Both are easily treatable and reversible causes of pancytopenia with good prognosis [22]. The above studies seem to reflect the higher prevalence of nutritional anemia in Indian subjects and other developing countries.

The small sample size, lack of a control group, and potential confounding variables are limitations of this study. This study could be a valuable resource for clinicians and researchers interested in hematological disorders and their clinical presentations.

## Conclusions

Pancytopenia is a common hematological entity, and it is a feature of various momentary illnesses or severe illnesses. It should be suspected when a patient presents with unexplained anemia, recurrent or prolonged infection, and bleeding tendency.

As observed in our study and various studies, especially in India, numerous causes of pancytopenia are reversible and treatable. Early and accurate diagnosis with intervention may be lifesaving and will definitely decrease the morbidity and mortality in these patients. Thus, detailed clinical study and hematological investigation along with bone marrow examination of patients with pancytopenia should be carried out, which can help in the proper identification of the underlying cause. Although rare, iron deficiency can present as pancytopenia and it should be kept as a differential diagnosis if common causes are ruled out, especially in India.

## References

[1] Miller DR (1994). Blood disease of infancy and childhood.

[2] Kumar SB, Arnipalli SR, Mehta P, Carrau S, Ziouzenkova O (2022). Iron deficiency anemia: efficacy and limitations of nutritional and comprehensive mitigation strategies. Nutrients.

[3] Schloesser LL, Kipp MA, Wenzel FJ (1965). Thrombocytosis in iron-deficiency anemia. J Lab Clin Med.

[4] Tichelli A, Gratwohl A, Speck B (1992). Iron-deficiency anemia: diagnosis and therapy. Schweiz Med Wochenschr.

[5] Lopas H, Rabiner SF (1966). Thrombocytopenia associated with iron deficiency anemia: a report of five cases. Clin Pediatr (Phila).

[6] Ganti AK, Shonka NA, Haire WD (2007). Pancytopenia due to iron deficiency worsened by iron infusion: a case report. J Med Case Rep.

[7] Tareen SM, Bajwa MA, Tariq MM, Babar S, Tareen AM (2011). Pancytopenia in two national ethnic groups of Baluchistan. J Ayub Med Coll Abbottabad.

[8] Tilak N, Jain R (1999). Pancytopenia - a clinical haematological analysis of 77 cases. Indian J Pathol Microbiol.

[9] Nanda A, Basu S, Marwaha N (2002). Bone marrow trephine biopsy as an adjunct to bone marrow aspiration. J Assoc Physicians India.

[10] Pozdniakova ZN (1955). [A case of multiple congenital abnormalities in infant, with diagnostic difficulty]. Sov Med.

[11] Kumar R, Kalra SP, Kumar H, Anand AC, Madan M (2001). Pancytopenia-a six-years study. J Assoc Physicians India.

[12] Knodke K, Marwah S, Buxi G, Vadav RB, Chaturvedi NK (2001). Bone marrow examination in cases of pancytopenia. J Academy Clin Med.

[13] Kuku I, Kaya E, Yologlu S, Gokdeniz R, Baydin A (2009). Platelet counts in adults with iron deficiency anemia. Platelets.

[14] Christensen RD, Liechty KW, Koenig JM, Schibler KR, Ohls RK (1991). Administration of erythropoietin to newborn rats results in diminished neutrophil production. Blood.

[15] McDonald TP, Clift RE, Cottrell MB (1992). Large, chronic doses of erythropoietin cause thrombocytopenia in mice [see comments]. Blood.

[16] Ishtiaq O, Baqai Hz, Anwer F, Hussai N (2004). Patterns of pancytopenia patients in a general medical ward and a proposed diagnostic approach. J Ayub Med Coll Abbottabad.

[17] Anita P, Vijay D (2013). Clinico-hematological analysis of pancytopenia: a bone marrow study. National J Laboratory Med.

[18] Varma A, Lokwani P, Malukani K, Gupta S, Maheshwari P (2018). Study of hematological profile of adults presenting with pancytopenia in a tertiary care hospital of central India. Med J DY Patil Vidyapeeth.

[19] Mansuri B, Thekdi KP (2017). A prospective study among cases of the pancytopenia on the basis of clinic-hematological analysis and bone marrow aspiration. Int J Res Med Sci.

[20] Karami H, Naderisorki M, Ghasemi M, Ghazaiean M (2021). Pancytopenia as a presentation of iron deficiency: a case report. J Pediatr Rev.

[21] HA Elhashmi (2019). Pancytopenia in 7 year-old child with severe iron deficiency anemia: case report. Int J Sci Res.

[22] Verma J, Sud R (2019). Clinico haematological profile in patients of pancytopenia and role of nutritional deficiencies as important aetiological and preventable factor in causing pancytopenia in India. Exp Hematol.

